# Adjusting for comorbidity in incidence-based DALY calculations: an individual-based modeling approach

**DOI:** 10.1186/s12874-020-00987-z

**Published:** 2020-05-06

**Authors:** Scott A. McDonald, Juanita A. Haagsma, Alessandro Cassini, Brecht Devleesschauwer

**Affiliations:** 1 Centre for Infectious Disease Control, National Institute for Public Health and the Environment (RIVM), PO Box 1, 3720 BA Bilthoven, Netherlands; 2 Department of Public Health, Erasumus MC University Medical Centre, PO Box 2040, 3000 CA Rotterdam, Netherlands; 3 Infection Prevention and Control Global Unit, World Health Organization, 20, Av Appia, CH-1211, Geneva 27, Switzerland; 4 Department of Epidemiology and Public Health, Sciensano, Rue Juliette Wytsmanstraat 14, 1050 Brussels, Belgium

**Keywords:** Multimorbidity, Individual-based modelling, Disability-adjusted life-years, YLD

## Abstract

**Background:**

The co-occurrence of two or more medical conditions in the same individual is not uncommon. If disability-adjusted life year (DALY) calculations are carried out for each condition separately, multimorbidity may lead to an overestimation of the morbidity component, the Years Lived with Disability (YLD). Adjusting for comorbidity may be straightforward if all symptoms have same onset and duration; however, when the comorbid health states occur at different time points, an analytical solution to the comorbidity problem becomes more complex. The aim of this study was to develop an individual-based modelling (IBM) approach to adjust incidence-based disease burden estimation for multimorbidity that allows simulating hypothetical individuals and tracking their disease history, including possible comorbidities, over time.

**Methods:**

We demonstrated the IBM approach using an example of external comorbidity, i.e., colon cancer comorbid with healthcare-associated pneumonia (HAP) and by assuming an independent multiplicative model. First, each cumulative progression probabilities were converted to a daily transition probabilities. Second, disability weights for simultaneously experienced health states and duration in each health state were determined. Third, YLD, adjusted for comorbidity, was calculated at every time step. We simulated a cohort of 1000 colorectal cancer patients aged 65 years. Ninety-five percent uncertainty intervals around median YLD values were estimated by Monte Carlo methods.

**Results:**

The median estimated YLD per 1000 cases (due to both cancer and HAP) adjusted for co-morbidity was 545 YLD/1000 (95% interval: 513–585). The impact of not adjusting disability weights for co-existent health states varied from minimal to small; YLD for colorectal cancer would be overestimated only slightly – by 1.6 YLD/1000 – by not adjusting for concurrent HAP. YLD for those HAP patients who have concurrent early-stage colorectal cancer would be overestimated by 2.3 YLD/1000.

**Conclusions:**

The computation of disease burden in the presence of multimorbidity using the incidence-based DALY approach can be handled through IBM. Our approach can be extended to other, more complicated multimorbidity scenarios which are responsible for a high current global disease burden, such as tuberculosis and HIV infection.

## Background

The co-occurrence of two or more medical conditions in the same individual is not uncommon, especially in the elderly [1]. In the medical literature, this phenomenon is often termed multimorbidity. When the focus is on an index disease, the presence of co-existing condition(s) – whether or not these are causally related to the index disease or occur independently – is typically referred to as comorbidity [2]. If disability-adjusted life year (DALY) calculations are carried out for each condition separately, multimorbidity may lead to an overestimation of the morbidity component, the Years Lived with Disability (YLD). Assume, for instance, a patient with simultaneous moderate osteoarthritis of the shoulder (disability weight (DW) = 0.117 [3];) and moderate chronic obstructive pulmonary disease (COPD)(DW = 0.225 [3];). Without adjusting for multimorbidity, the DW for this patient would implicitly be calculated as the sum of both DWs, i.e., 0.117 + 0.225 = 0.342. However, assuming that DWs can simply be summed to capture the level of disability experienced by a patient with multimorbidity, is not necessarily correct [4, 5]. Furthermore, additivity could lead to a multimorbid DW that is larger than one, i.e., a situation “worse than death”.

Various other methods have been described to account for multimorbidity and overcome the limitations of the additive approach [5]. In the maximum limit or worst case approach, the multimorbid DW is set equal to the highest DW of the individual conditions. For our patient, this would result in a multimorbid DW of 0.225; i.e., that of COPD. In the multiplicative approach, the multimorbid DW is calculated as: 1 − ∏_*i*_(1 − *DW*_*i*_), with DW_*i*_ the individual DW. For our patient, this would result in a DW of 1 − (1–0.117)(1–0.225) = 0.316. Assuming that the multiplicative approach is approximately correct and that the additive approach overestimates experienced disability, it is easy to see that the degree by which disability, and thus YLD, is overestimated using the additive approach grows with the severity of either comorbid condition (the difference between the two approaches is greatest when the DWs approach 1.0). Underestimation of true experienced severity by both the additive and multiplicative approaches is also possible.

### Dealing with comorbidity: prevalence vs. incidence-based models

In addition to a model for combining DWs for a given patient, dealing with comorbidity also requires epidemiological data on the co-occurrence of disease. Ideally, this information should come from a population-based health survey. However, due to the large number of possible causes of ill health (if there are *h* diseases/conditions that can co-occur, then there are *2*^*h*^*– 1* occurrence/co-occurrences possible), it is practically impossible to measure all possible diseases and conditions in a population sample [6]). In absence of such information, a pragmatic solution was proposed in the GBD 2010 study by introducing prevalence-based YLDs, which made it feasible to correct for multimorbidity. The GBD authors used microsimulation methods to estimate the prevalence of multimorbidity from the prevalence of the individual conditions, within each age and sex stratum [7, 8].

The original formulation of the DALY metric, however, prescribed an incidence perspective for calculating YLDs [9]. Using this perspective, YLDs measure future health losses due to current exposures, and is therefore often the preferred approach for quantifying the burden of infectious diseases [10, 11]. However, accounting for multimorbidity in incidence-based disease models is more complicated – because the onset times and duration of diseases/conditions do not normally coincide – and no practical solutions have been provided.

Multimorbidity in disease models may occur in various forms. We propose a pragmatic classification, into *internal* and *external* comorbidity. Internal comorbidity refers to co-occurrence of health states within the same disease model, while external comorbidity refers to co-occurrence of multiple diseases (i.e., not within the same model). For instance, the co-occurrence of different health states associated with the same infectious disease would be considered internal comorbidity, while the co-occurrence of the health states of the infectious disease with the health states of a non-communicable disease occurring in the same patient, would be considered external comorbidity. The distinction between both depends on the definition of what comprises a disease model (for instance, both the infectious and non-communicable disease in our example can have a joint risk factor, and could thus be combined in a risk factor-based disease model), and is as such arbitrary. Nonetheless, it is an operational definition that is useful to demonstrate possibilities and corresponds to current literature on disease burden calculations [12].

The main objective of this paper is to develop a method to adjust incidence-based disease burden estimation for multimorbidity that can be applied to two or more co-existing health states. To correctly compute YLD in the presence of comorbidity, we propose an individual-based modelling approach. We illustrate this approach by calculating YLD for a worked example of ‘external comorbidity’, i.e., colon cancer complicated by healthcare-associated pneumonia (HAP). Although years of life lost (YLL) is the second necessary component of the DALY measure, in the current paper we restrict to YLD because distinct analysis strategies are required to partition YLL among competing mortality risks in the case of multimorbidity [13].

## Methods

### Approach for dealing with comorbidity using incidence -based DALY

**Fig. 1:**
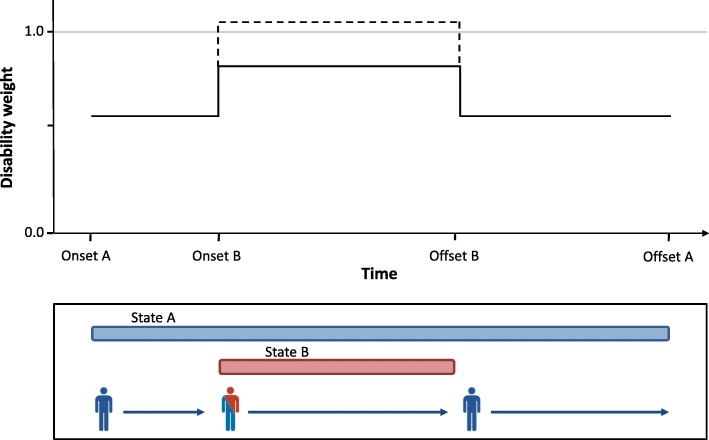
Hypothetical time course of experienced departure from full health (disability weight) for an individual in two health states with delayed onset. The dashed line indicates the additive approach, and the solid line indicates the multiplicative approach to experienced disability

Within a given disease model, multiple health states may occur in sequence (for instance, an acute followed by a chronic disease stage) or in parallel – with the latter being either mutually exclusive (for instance, multiple severity levels), or not (for instance, multiple symptoms). Using the terminology introduced before, this may give rise to a situation of internal comorbidity. Figure 1 gives a schematic representation of how health states can overlap in time for a single individual and the consequence for experienced disability. If, continuing with the example above, during the entire time that a patient suffered from COPD he/she also had osteoarthritis, then YLD for osteoarthritis would be overestimated by 8% (1–0.342/0.316) if the co-existence of COPD was assumed to have an additive effect on severity, compared with adjustment using the multiplicative approach.

**Fig. 2:**
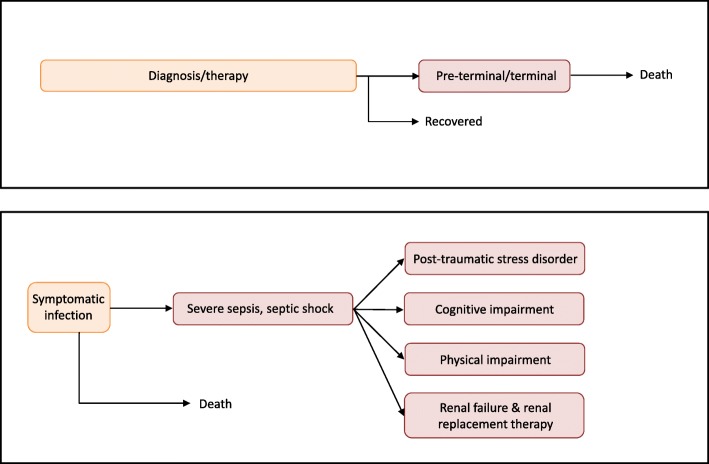
(Upper panel) Simplified clinical progression pathway for colorectal cancer. Health states are indicated by boxes, with relative box lengths indicating average duration in that health state. (Lower panel) Clinical progression pathway for healthcare-associated pneumonia infection (adapted from [14, 15]), showing the six relevant health states as filled boxes

By definition, comorbidity occurs for health states that overlap in time and that are not mutually exclusive. Adjusting for comorbidity may be straightforward if all symptoms have same onset and duration. For instance, following severe sepsis due to healthcare-associated pneumonia (HAP) infection (Fig. 2), one may develop one or more lifelong disabilities. Indeed, in such cases, comorbidity occurs throughout the rest of the patient’s life (in our example: post-traumatic stress disorder (PTSD), cognitive impairment, physical (mobility) impairment, and renal failure). Therefore, to compute YLD the model requires only the probabilities of disease occurrence and possible correlations, the disability weight of each health state, and a choice to be made on how to appropriately combine disability weights.

When the comorbid health states occur at different time points, an analytical solution to the comorbidity problem becomes more complex, especially if one wants to consider variability and/or uncertainty in the calculations. Indeed, the adjustment requires knowledge on the specific time points when multiple conditions co-exist.

To address this problem, we adopt an individual-based model (IBM) approach, which allows the simulation of a hypothetical cohort of individuals and the tracking of their disease history, including possible comorbidities, over time. We demonstrate this approach using an example of external comorbidity, i.e., colorectal cancer complicated by HAP.

### Example IBM for external comorbidity

There are two health states standardly assumed within the clinical progression pathway for cancers that respond to treatment: *Diagnosis* and *Control/management*. For non-responders, there are additional two states: *Pre-terminal* (or *metastatic*) and *Terminal*. We simplified the pathway to two states: *Diagnosis/therapy* and *Pre-terminal/terminal* states (Fig. 2). For colorectal cancer, the probability of cure is age-dependent, but for simplicity we set this parameter to a single value, 56% [16]. This means the probability of death from cancer was (100–56%) = 44%. In addition, we did not consider possible sequelae occurring among cured patients.

The risk of acquiring HAP was restricted to the *Diagnosis/therapy* state only, when cancer patients would be initially admitted to hospital. This was not easily obtainable from the literature; for our worked example we set this parameter to 12%, which is the prevalence of all HAIs among patients in the specialty *Transplant/cancer surgery* from a point prevalence survey in the European acute-care setting, 2011–2012 [17]. This is higher than earlier reports of HAP incidence (e.g., crude rate of 6.1/1000 discharges [18];). Table 1 details all colorectal cancer disease model parameters.

**Table 1:** Results of individual-based modelling of worked example of external comorbidity

Disease [− health state]	Adjusted YLD/1000 cases (95% UI)	Unadjusted YLD/1000 cases (95% UI)	Overestimation of YLD (95% UI)	Difference in YLD/1000 cases
Colorectal cancer (All health states)	471.7 (447.8–496.8)	473.3 (449.2–498.9)	0.3**%**	1.6
**–** Diagnosis/therapy only	309.4 (291.8–329.9)	310.5 (292.8–331.1)	0.4%	1.1
**–** Pre-terminal/ terminal only	163.5 (147.4–178.7)	164.0 (148.0–179.2)	0.3%	0.5
HAP co-existent with colorectal cancer (All health states)	73.0 (51.4–98.1)	75.3 (52.8–102.0)	3.0%	2.3
**–** Symptomatic infection	0.4 (0.3–0.5)	0.4 (0.3–0.5)	9.9%	0.0
**–** Severe sepsis, septic shock	1.1 (0.8–1.5)	1.4 (0.9–1.9)	25.5%	0.3
**–** PTSD	17.4 (8.1–28.8)	18.1 (8.4–29.8)	3.9%	0.7
**–** Cognitive impairment	14.4 (8.7–21.1)	14.8 (8.9–21.7)	3.0%	0.4
**–** Physical impairment	36.2 (28.0–45.4)	36.7 (28.4–46.0)	1.4%	0.5
**–** Renal failure & renal replacement therapy	4.2 (0–13.8)	4.5 (0–4.5)	7.9%	0.3

*Note.* Results compare Years Lived with Disability (YLD) per 1000 cases for colorectal cancer adjusted and unadjusted for comorbidity due to healthcare-associated pneumonia (HAP), and also HAP (among patients with colorectal cancer), both adjusted and unadjusted for comorbidity, in terms of absolute and relative differences in YLD

*PTSD* post-traumatic stress disorder, *UI* uncertainty interval

We adopted a previously published clinical pathway progression model for HAP [14], which consists of the health states *Symptomatic infection* followed by either, recovery, *Severe sepsis* or death. Patients who had progressed to *Severe sepsis* can develop the life-long (and non-mutually exclusive) sequelae *PTSD*, *Cognitive impairment,* and/or *Renal failure*. Following *Severe sepsis,* all patients develop *Physical impairment* (Fig. 2 and Table A2). Although intervals/ranges were available for many model parameters, for simplicity we used only point estimates.

#### Progression probabilities

The probability of each of these combinations of health states is the product of the probability of observing (or not) of each individual health state – at least, if independence between health states is assumed. We modelled comorbidity assuming an independent multiplicative model, i.e., the probability of simultaneously experiencing a combination of health states is the product of the probabilities of experiencing each of the individual health states, regardless if the health states are part of the clinical progression pathway for the same disease/agent or are part of different disease pathways.

Because our IBM uses a time step of 1 day, we first needed to convert each cumulative progression probability to a daily rate. For example, in the HAP disease model, the health state *Severe sepsis* develops following *Symptomatic infection* with a cumulative probability of 0.39 (Table A2), meaning that 39% of all individuals progress to this health state. We converted this cumulative probability to a daily rate, based on the mean duration in the *Symptomatic infection* health state of 0.025 years (=9.1 days), using the standard formula:

$$ {r}_{\mathrm{sepsis}}=-\log \left(1-0.39\right)/9.1 $$

and then to a daily transition probability for use in the IBM:

$$ 1-\exp \left(-{r}_{\mathrm{sepsis}}\right) $$

The daily transition probability for this example is 0.0529. Point estimates for the cumulative probabilities of developing all other health states were similarly converted to daily transition probabilities. The cumulative probability of cancer patients acquiring HAP infection during the *Diagnosis/therapy* state, 0.12, was converted in the same way, yielding a daily probability of HAP of 0.00016.

#### Determining disability weights for simultaneously experienced health states

We define the disability weight for a time step as a function of all of the health states experienced by the individual at that time step. For the adjusted method, we use the multiplicative approach (i.e.: 1 − ∏_*i*_(1 − *DW*_*i*_)) as described previously [5, 19]. To permit comparison to a measure of YLD unadjusted for comorbidity, we adopted the published disability weights for each health state instead of adjusting weights according to the formula in the previous sentence, and so effectively assumed additivity in experienced disability for co-existing health states.

Disability weights for the individual health states for colorectal cancer were obtained from Salomon et al. [3], and weights for HAP were taken from Burden of Communicable Diseases in Europe (BCoDE) reports and other sources [14, 15, 20] (Tables A1 and A2).

#### Duration in each health state

For colorectal cancer, the mean duration of the *Diagnosis/therapy* state was set to 1.08 years [16]; as the median survival time for patients who die from this cancer is 1.6 years [16], the mean duration of the *Pre-terminal/terminal* state was set to (1.6–1.08) = 0.52 years)*.* Mean durations for the various HAP health states were taken from the BCoDE project reports [14, 15] (see Table A2).

#### YLD computation

YLD, adjusted for comorbidity, was calculated at every time step *i* as in the following, where *Z*_*i*_ is the maximum number of co-existent health states at time step *i* (which can vary as the prevalence and nature of multimorbidity is time-dependent), *n*_*s,i*_ is the number of individuals in state *s* at time step *i*, and *DW*_*adj,i*_ is the disability weight at time step *i* adjusted using the multiplicative method (above):

$$ {DW}_{ad\mathrm{j}.i}=1-{\prod}_s\left(1-{DW}_s\right) $$

To allocate disability among the set of health states experienced by an individual at time *i*, *DW*_*adj,i*_ is proportionally redistributed to these health states:

$$ {DW}_{adj,s.i}={DW}_{adj.i}\ast \frac{DW_{s,i}}{\sum \limits_{s=1}^Z{DW}_{s,i}} $$

Adjusted YLD is then calculated as:

$$ {YLD}_{adj,i}=\sum \limits_{s=1}^{Z_i}{n}_{s,i}\times {DW}_{adj,s,i} $$

YLD, unadjusted for comorbidity, was similarly computed at each time step *i*, except the unadjusted disability weight, *DW*, was used:

$$ {YLD}_{unadj,i}=\sum \limits_{s=1}^{Z_i}{n}_{s,i}\times {dw}_s $$

#### Simulation procedure and R code

We simulated a cohort of 1000 colorectal cancer patients aged 65 years; residual life expectancy at this age was fixed at 20 years. Onset of cancer disease burden was defined as the moment of diagnosis. The onset of possible HAP infection was stochastically simulated for each individual as a draw from a Bernoulli distribution at each time-step during the *Diagnosis/therapy* health state only. From the onset of HAP, random draws from the relevant distributions were made at each time step to simulate progression from *SI* to *Sepsis* and from SI to death. Progression to one or more of the four long-term sequelae could occur after the offset of *Sepsis*. Note that in the IBM approach HAP health states can also be experienced simultaneously; for instance *PTSD* and *Physical impairment*. All patients progressed through the colorectal cancer clinical pathway; random Bernoulli draws were made to determine if a patient was cured, or progressed to the *Pre-terminal/terminal* health state and then to death (Fig. 2). The principal outcome was defined as the absolute difference between comorbidity- unadjusted and adjusted and YLD among the entire cohort, per disease, and per health outcome; the relative difference was also computed.

Ninety-five percent uncertainty intervals – accounting for stochasticity – around median YLD per 1000 cases were estimated by Monte Carlo methods; namely, by repeating the simulation 500 times and calculating the median and 2.5th and 97.5th percentiles of the resulting distribution of simulated YLD values. Simulations were conducted in the R statistical programming environment, version 3.5.1 [21]. R code to implement both IBM simulations is provided in Additional file 1.

### Sensitivity analysis

As there is uncertainty regarding the selected values of all model parameters, although largely taken from previous research, it is of interest to explore to what degree the choice of parameter value impacts on the results, by considering alternative values to the point estimate. We therefore carried out one-way sensitivity analyses for each of five parameters (the progression probabilities to *Sepsis* and to death following *SI* with HAP, the risk of developing HAP among patients with colorectal cancer, the progression probability from *Diagnosis/therapy* to *Pre-terminal/terminal* health state, and the total duration in all health states of colorectal cancer), by testing the effect of a parameter value either 20% lower or 20% greater than the ‘baseline’ value (i.e., the point estimate), and re-running the simulation.

## Results

The median estimated cumulative total YLD per 1000 cases (due to both cancer and HAP) – representing the future disease burden ‘assigned’ to the affected cohort of 1000 colorectal cancer patients under the incidence-based DALY approach – adjusted for co-morbidity was 545.3, and was reasonably stable over 500 simulations (95% uncertainty interval of 513.1–584.6).

**Fig. 3:**
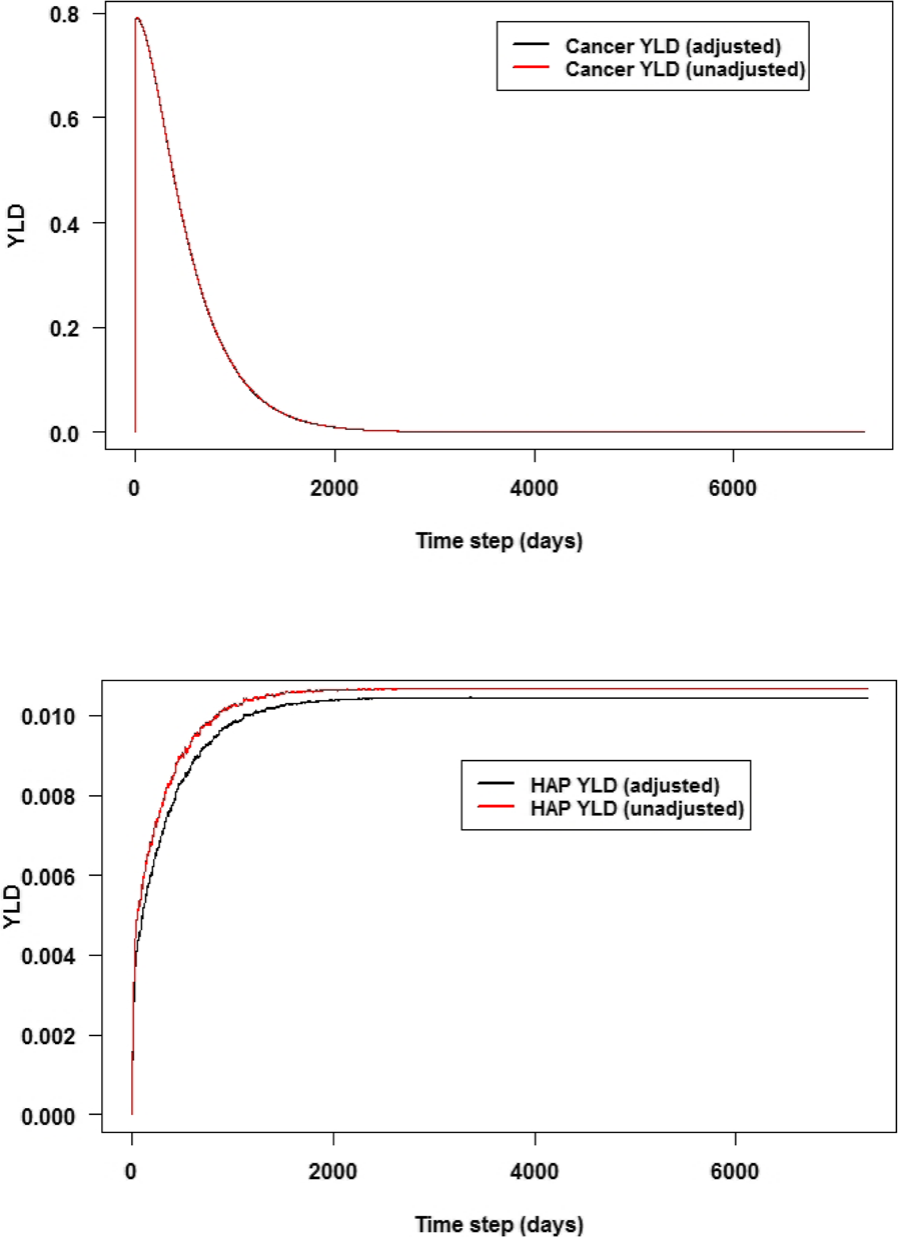
(Upper panel) Years lived with disability (YLD) per 1000 cases per day, for colorectal cancer over time, unadjusted or adjusted for co-occurrence with healthcare-associated pneumonia (HAP), for a simulated cohort of cancer patients from age 65 to 85 years. (Lower panel) Comorbidity-adjusted YLD per 1000 cases per day, for HAP, for 120 HAP infections occurring among a simulated 1000 patient cohort. Adjusted YLD in both panels is overlaid with YLD computed using the unadjusted approach; note that the two series in the upper panel almost completely overlap. Values represent means over 500 simulations

Cumulative total YLD was overestimated by 4.1 per 1000 cases (relative measure: 0.7%) if no disability adjustment is made (median YLD of 549.4 for the unadjusted measure; see Fig. A1). Figure 3 shows (highly overlapping) estimated YLD for colorectal cancer over time comparing unadjusted and adjusted variants of the model, and estimated YLD for HAP (as occurring in cancer patients) both adjusted and unadjusted for comorbidity with cancer. Prevalence over time, for all health states of both cancer and HAP is depicted in Fig. A2.

Table 1 compares comorbidity-adjusted YLD with YLD computed when not adjusting for disease co-existence, for each disease and health state separately. The morbidity burden computed for cancer among a cohort of colorectal cancer patients in which HAP was co-existent was slightly higher (1.6 YLD/1000 cases; relative 0.3%) when not adjusting for comorbidity (median YLD of 471.7 and 473.3, for unadjusted and adjusted measures, respectively). The morbidity burden attributable to HAP among a cohort of cancer patients in which HAP co-occurred was also slightly overestimated, by 2.3 YLD/1000 (relative 3.0%), if disability weights were not adjusted for comorbidity (median YLD of 73.0 and 75.3 for unadjusted and adjusted measures, respectively). This overestimation was mostly driven by the *PTSD* and *Physical impairment* health states (0.7 and 0.5 higher YLD/1000 for unadjusted measures, respectively; Table 1).

The results of the one-way sensitivity analysis indicated that the selected parameter value for the five parameters explored only minimally affected the outcome measure (the difference in absolute YLD per 1000 cases between adjusted and unadjusted models (Fig. A3). The largest impact was observed for the progression probability from symptomatic infection with HAP to severe sepsis, which when set to 20% lower than the baseline value, a difference of 2.5 in absolute YLD/1000 cases between model variants was observed.

## Discussion

We have demonstrated that individual-based modelling offers a practical means to deal with multimorbidity in the calculation of YLD using the incidence-based approach to disease burden estimation. The IBM method proved useful, firstly for demonstrating that failing to adjust for a single comorbid condition can lead to overestimation of disease burden, and secondly for providing a practical implementation for ‘real-world’ disease burden estimation using the incidence-based approach. This means that given suitable and unbiased data on the distribution of multimorbidity (which may be challenging in itself, as is the case for all data required for DALY estimation), the totality of disease burden in a given patient population can be correctly computed. With slight modifications, the IBM code can be used for other conditions (such as injuries), and to address issues such as individual heterogeneity in progression probabilities [22].

For our external comorbidity example, the impact of not adjusting disability weights for co-existent health states varied from minimal to small; YLD for colorectal cancer would be overestimated only slightly – by 1.6 YLD/1000 cases – by not adjusting for concurrent HAP. YLD for those HAP patients who have concurrent early-stage colorectal cancer would also be slightly overestimated, by 2.3 YLD/1000 cases. Importantly, failing to adjust for internal comorbidity also leads to an overestimated burden attributable to HAP (because of potentially overlapping long-term sequelae), irrespective of the presence of an underlying condition such as cancer.

The impact of adjustment on the disease burden among patients with multimorbidity is expected to be very small in the case of internal comorbidity. This is because the distribution of risk over the health states of a given disease potentially occurring simultaneously tends to be heavily skewed; most states have a very low risk of occurrence (at least for infectious diseases [23]). In our worked example, the long-term sequelae of HAP constitutes a case of internal comorbidity, as an individual can occupy one or more of the four health states following *Severe sepsis* simultaneously.

Previous approaches for adjusting health burden measures for comorbidity include regression analysis to decompose the effects of multimorbidity on HRQoL [24, 25]. Similar decomposition methods have been used to examine the contribution made by causes of disability to differences in healthy life expectancy [26, 27] or disability prevalence [28]. Unlike the classical DALY approach, these methods allow for a “background” burden, i.e., disability in subjects without a reported disease. Unfortunately, decomposition methods only permit the study of the contribution of multiple causes to overall health; they do not allow one to calculate a DW for an arbitrary set of conditions. While adjusting DWs for co-occurring health states has straightforward application within prevalence-based DALY estimation [7, 19], and recent work has dealt with internal co-morbidity within the incidence-based DALY framework [12], to our knowledge there has been no previous efforts to develop a general-purpose method for incidence-based DALYs involving external co-morbidity using individual-based modelling.

### Limitations

Our approach used a multiplicative approach to combine DWs for temporally overlapping health states. Although this approach has the desired mathematical property of not resulting in comorbid DWs higher than 1, there is little empirical evidence on the validity of this model. For purposes of the worked example, we did not consider uncertainty in any parameter values assumed (we used point estimates only), but the one-way sensitivity analysis indicated that the conclusions held given changes of plus or minus 20% of the baseline parameter value.

In addition to an appropriately adjusted multimorbid DW, calculating YLDs also requires data on the prevalence or incidence of the multimorbid condition. In the GBD studies, the prevalence of multimorbidity was estimated by assuming independence between the prevalence of the individual diseases [7, 8]. Assuming independence for our worked example would mean that the prevalence of the given multimorbidity would equal the product of the prevalence of colorectal cancer and the prevalence of HAP, which would appear to be implausible as the risk of HAP is higher for ICU than non-ICU patients [29], for instance. This could lead to an overestimation of the burden of the individual diseases. Murray et al. [7] argue that the error associated with the independence assumption is minimal when this assumption is applied within each specific age–sex group. However, there is little evidence to support this assumption. Data on the correlations between occurrence of different conditions, e.g., quantified using odds ratios [19], would help to assess this independence assumption.

Because we restricted our simulation to YLD, we did not consider how to handle a potentially increased mortality rate if a person has two or more co-morbid conditions. This primarily would have an impact on the computation of YLL, but could also affect YLD by truncating the time lived with long-term sequelae. Similarly, our approach does not currently handle the situations in which a second condition influences the duration of one or more health states of the first, or in which a second condition influences the clinical pathway of the first (i.e., by incurring long-term disability; for instance a lower respiratory tract infection worsening COPD). However, the IBM could be extended to take such interdependencies into account.

Lacking suitable data on the rate of HAP among cancer patients undergoing diagnosis/therapy, the parameter value we selected for the cumulative risk of HAP for patients in this health state is meant to illustrate the difference between adjustment/non-adjustment only. However, sensitivity analysis indicated that results were only slightly dependent on the precise value chosen. Finally, the impact of comorbidity adjustments is variable, and depends on the epidemiological context and the adjustment methodology chosen [19]). It is likely that such adjustments may have a greater impact on patient-level compared with population-level burden estimates – a significant difference may be seen in selected patient groups such as the elderly with a high prevalence of multi-morbidity but not when considering the national population – and consequently have a larger impact on health economic evaluations.

For didactic purposes, our simulation contained many simplifications compared with DALY computation in practice: only two diseases, probability of cure was independent of age, residual life expectancy was set at 20 years for every cohort member, and mortality rates were assumed unaffected by the presence of comorbidity. Nevertheless, the IBM approach can incorporate all of these complexities.

For analysts using the incidence-based DALY approach, we suggest implementing the proposed methods for certain diseases (for which suitable comorbidity prevalence data – in particular, data on the co-occurrence probabilities of comorbid conditions – are available) in parallel to routine burden calculation activities, so that results using the proposed approach can be compared to the findings obtained using standard methodology. This will assist in ascertaining the expected magnitude of differences in YLD estimates realised in ‘real-world’ practice between comorbidity-adjusted and unadjusted approaches, and whether there are any implications for disease ranking in terms of burden that may be used by policy-makers for prioritisation.

## Conclusions

The computation of disease burden in the presence of multimorbidity using the incidence-based DALY approach can be handled through IBM. In our worked example, adjusting for comorbidity produced YLD estimates that only slightly differed from unadjusted estimates. We purposely demonstrated our approach a using simple, yet realistic scenario. Our approach can be extended to other, more complicated multimorbidity scenarios which are responsible for a high current global disease burden, such as tuberculosis and HIV infection [30]. Further investigation of the current approach using such diseases is required to determine if comorbidity adjustment for disease burden computation will have relevance for public health decision-making.

## Supplementary information

**Additional file 1.** Supplementary materials. File contains supplementary tables of disease model parameters, figures showing results of the worked example, and R code to run the simulations.

## Data Availability

All data are supplied in the main article and supplementary materials.

## Abbreviations

DALY: Disability-adjusted life year
YLD: Years Lived with Disability
IBM: Individual-based modelling
HAP: Healthcare-associated pneumonia
DW: Disability weight
COPD: Chronic obstructive pulmonary disease
BCoDE: Burden of Communicable Diseases in Europe
PTSD: Post-traumatic stress disorder
