# Supplementary material for: Adjusting for comorbidity in incidence-based DALY calculations: an individual-based modeling approach
Source: BMC Med Res Methodol. 2020 May 6;20:100. doi: 10.1186/s12874-020-00987-z (PMC7201540; doi:10.1186/s12874-020-00987-z)
Supplement: Supplementary file 1 — Additional file 1. Supplementary materials. File contains supplementary tables of disease model parameters, figures showing results of the worked example, and R code to run the simulations. [file 12874_2020_987_MOESM1_ESM.pdf]

ADDITIONAL FILE 1

Supplementary Tables and Figures

For the paper entitled:

Adjusting for comorbidity in incidence-based DALY calculations: an individual-based modeling approach

Tables of parameters for clinical progression pathways of the example diseases

Table A1. Simplified disease model for colorectal cancer

**Health state**	**Progression probability**	**Disability weight (DW)**	**Duration (years)**	**Reference**
Diagnosis/therapy	-	0.288	1.08	(DW) Salomon et al., 2015<br>(D) Soerjomataram et al., 2012
Pre-terminal/terminal	0.44	0.540	0.52	(DW) Salomon et al., 2015<br>(D) Soerjomataram et al., 2012
Death following Pre-terminal/terminal	1.0	-	-	

Table A2. Disease model for healthcare-associated pneumonia (Cassini et al., 2016; Colzani et al., 2017). RLE = remaining life expectancy. Point estimates only were used in the simulation, and therefore as-published 95% intervals or ranges for parameter values are not shown.

**Health state**	**Progression probability**	**Disability weight (DW)**	**Duration (years)**	**Notes**
Symptomatic infection	-	0.125	0.025	
Severe sepsis, septic shock	0.39	0.655	0.0315	
Death following symptomatic infection	0.035	-	-	
Post-traumatic stress disorder	0.17	0.088	RLE	
Cognitive impairment	0.29	0.043	RLE	*Midpoint of published range chosen*
Physical impairment	1.0	0.032	RLE	*Midpoint of published range chosen*
Renal failure and renal replacement therapy	0.011	0.259	RLE	*Midpoint of published range chosen*

Fig. A1. Cumulative years lived with disability (YLD) per 1,000 cases for both cancer and HAP in a simulated cohort of 1,000 cancer patients. Overlaid with cumulative YLD computed using the unadjusted approach.

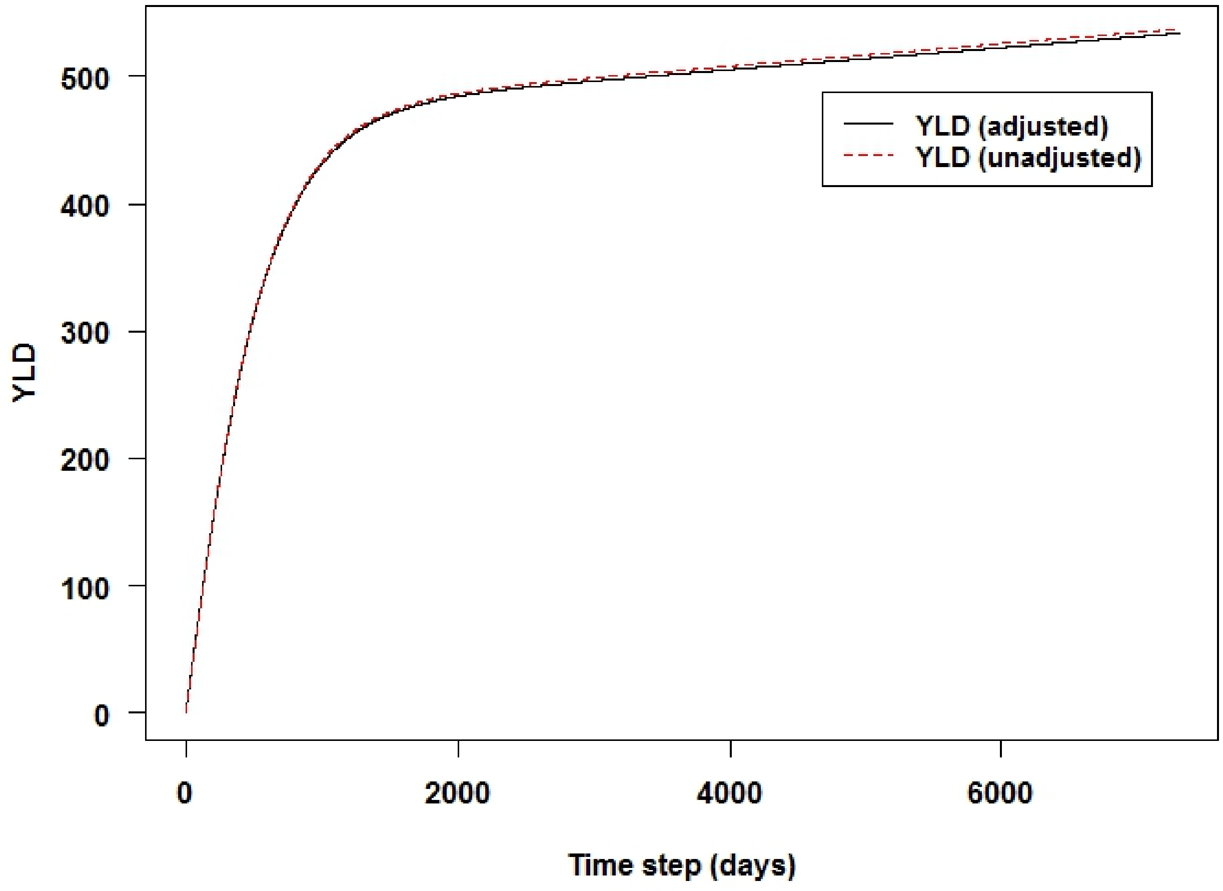

Fig. A2. Prevalence over time of all health states in both colorectal cancer and HAP disease models in a simulated cohort of 1,000 individuals with colorectal cancer. Time step is in days.

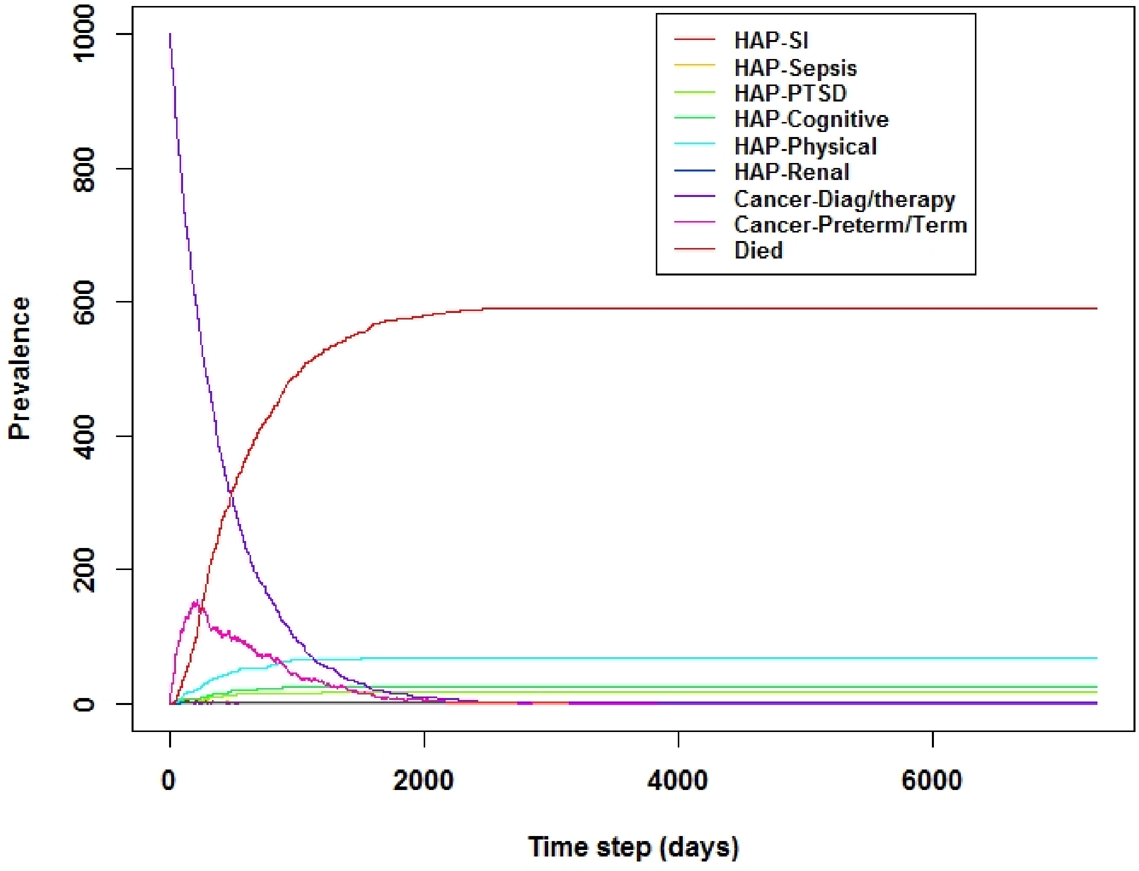

Fig. A3. Tornado plot showing the results of one-way sensitivity analysis conducted on five model parameters.

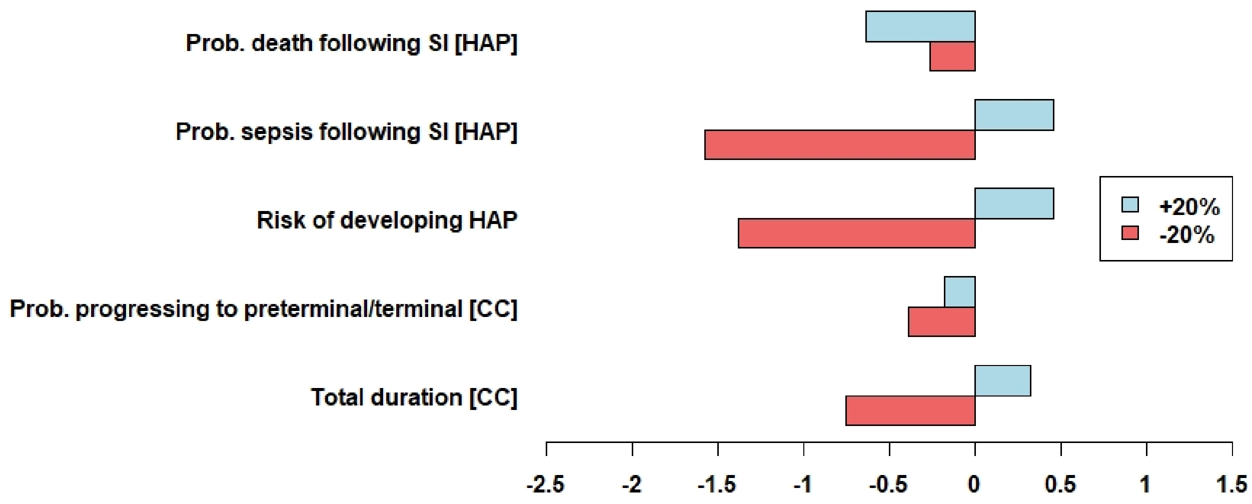

R code

```
### DALY individual based model

library(matrixStats)
library(plyr)

set.seed(101)


## functions

# Multiplicative assumption for combining dws
dw_multi <-
function(...) {
  dw <- unlist(list(...))
  1 - prod(1 - dw)
}

dw_add <-
function(...) {
  dw <- unlist(list(...))
  sum(dw)
}

get_yld <-
function(x) {
  sum(x$dw)
}

get_yld_unadj <-
function(x) {
  sum(x$dw.unadj)
}



#### colorectal cancer and HAP

## settings
nsim <- 500     # number simulations
n <- 1000       # number of individuals
s <- 365*20     # number of time steps (in days)
mean_age <- 65  # mean age of population (years); this is arbitrary

# indices 1-6 are for health states of HAP, 7-8 for cancer
dw1 <- 0.125    # symptomatic inf (SI)
dw2 <- 0.655    # sepsis
dw3 <- 0.088    # PTSD
dw4 <- 0.043    # cognitive impairment
dw5 <- 0.032    # physical impairment
dw6 <- 0.2585   # renal failure

dw7 <- 0.288    # diagn/therapy
dw8 <- 0.540    # preterm/term

dw_all <- c(dw1, dw2, dw3, dw4, dw5, dw6, dw7, dw8)

d1 <- (0.025*365)               # SI mean duration of 9.125 days
d2 <- (0.0315*365)              # Sepsis mean duration of 11.50 days
d3 <- (20-0.025-0.0315)*365     # mean duration of (20 years minus mean duration of previous states)
d4 <- (20-0.025-0.0315)*365
d5 <- (20-0.025-0.0315)*365
d6 <- (20-0.025-0.0315)*365

d7 <- (1.08*365)   # diag/therapy: 394.2 days
d8 <- (0.52*365)   # preterm/term: 189 days


# Daily risk of developing HAP during state 7 (Cancer-Diag)
rate_HAP <- -log(1-0.12)/d7    # convert per-patient cumul risk to daily rate - ECDC point prev of 12%
among all HAIs cancer surgery
r_HAP <- 1-exp(-rate_HAP)

ctp_12 <- 0.39                 # cumulative transition probability SI -> Sepsis
rate_12 <- -log(1-ctp_12)/(d1) #  daily exit rate 1->2 = 0.0542
tp_12 <- 1-exp(-rate_12*1)     # but, need to convert to day-level TP = 0.05272843
ctp_23 <- 0.17                 # cumulative transition probability Sepsis -> PTSD
ctp_24 <- 0.29                 # cumulative transition probability Sepsis -> Cogn Impair
ctp_25 <- 1.0                  # cumulative transition probability Sepsis -> Phys Impair
```

```
ctp_26 <- 0.011                # cumulative transition probability Sepsis -> Renal failure
ctp_1d <- 0.035                # cumulative mortality prob. following SI
rate_1d <- -log(1-ctp_1d)/(d1)
tp_1d <- 1-exp(-rate_1d*1)     # daily mort rate 1->3 = 0.00390

ctp_78 <- 0.44                 # cumulative transition probability Diag/therapy -> Preterm/term
rate_78 <- -log(1-ctp_78)/(d7) #  daily exit rate 7->8 = 0.00147
tp_78 <- 1-exp(-rate_78*1)     # but, need to convert to day-level TP = 0.00147
rate_8d <- 1/d8                # daily mortality rate is 0.00527
tp_8d <- 1-exp(-rate_8d*1)     # convert to day-level TP

# Recovery/exit probabilities based on expected rate leaving state (1/duration in state)
rate_1r <- ((1/d1) - rate_12 - rate_1d)
rp_1 <- 1-exp(-rate_1r*1)      # recovery probability SI -> R
rate_2r <- (1/d2)
rp_2 <- 1-exp(-rate_2r*1)      # exit probability Sepsis
rate_7r <- ((1/d7) - rate_78)
rp_7 <- 1-exp(-rate_7r*1)      # recovery probability Diag/therapy -> R (cure)



# Define IBM as function
sim <- function() {

 ## initialize; 1 row for each individual
 dfr <- data.frame(age = rep(mean_age,n),
                  hs1 = FALSE,
                  hs2 = FALSE,
                  hs3 = FALSE,
                  hs4 = FALSE,
                  hs5 = FALSE,
                  hs6 = FALSE,
                  hs7 = TRUE,
                  hs8 = FALSE,
                  exit_2 = FALSE,  # indicator that hs2 (Sepsis) has been exited
                  d = FALSE,       # 'Died' indicator
                  dw = dw7,
                  dw.unadj = dw7)
 yld <- numeric(s)                # for total YLD (both cancer & HAP)
 yld[1] <- get_yld(dfr)
 yld_unadj <- numeric(s);                       yld_unadj[1] <- get_yld_unadj(dfr)
 yld_cc <- numeric(s);                          yld_cc[1] <- sum(dfr$dw[dfr$hs7]*dfr$hs7)
 yld_cc_unadj <- numeric(s);                    yld_cc_unadj[1] <- sum(dfr$dw.unadj[dfr$hs7]*dfr$hs7)
 yld_hap <- numeric(s);                         yld_hap[1] <- 0
 yld_hap_unadj <- numeric(s);                   yld_hap_unadj[1] <- 0
 yld_cc_state <- matrix(0,nrow=2,ncol=s);       yld_cc_state[2,1] <- 0
 yld_cc_state[1,1] <- sum(dfr$dw[dfr$hs7]*dfr$hs7)
 yld_cc_state_unadj <- matrix(0,nrow=2,ncol=s); yld_cc_state_unadj[2,1] <- 0
 yld_cc_state_unadj[1,1] <- sum(dfr$dw.unadj[dfr$hs7]*dfr$hs7)
 yld_hap_state <- matrix(0,nrow=6,ncol=s);      yld_hap_state[,1] <- 0
 yld_hap_state_unadj <- matrix(0,nrow=6,ncol=s); yld_hap_state_unadj[,1] <- 0

 # Define variables useful for plotting
 prev <- matrix(0,nrow=s,ncol=9)     # 9th column for 'Dead'
 prev[1,] <- c(0,0,0,0,0,0,n,0,0)    # starting cohort is in 7th state


 ## run
 for (i in seq(2, s)) {
  ## indicator vectors; randomly determine HAP infection, progressions, deaths
  get_hs1 <- rbinom(n, 1, r_HAP) == 1
  get_hs2 <- rbinom(n, 1, tp_12) == 1
  get_hs3 <- rbinom(n, 1, ctp_23) == 1
  get_hs4 <- rbinom(n, 1, ctp_24) == 1
  get_hs5 <- rbinom(n, 1, ctp_25) == 1
  get_hs6 <- rbinom(n, 1, ctp_26) == 1
  get_d1 <- rbinom(n, 1, tp_1d) == 1
  recover_hs1 <- rbinom(n, 1, rp_1) == 1
  recover_hs2 <- rbinom(n, 1, rp_2) == 1

  get_hs8 <- rbinom(n, 1, tp_78) == 1
  get_d8 <- rbinom(n, 1, tp_8d) == 1
  recover_hs7 <- rbinom(n, 1, rp_7) == 1

  ## individuals may die if in origin state 1 (HAP-SI) or in state 8 (Cancer-Preterm/term) only
  has_1 <- dfr$hs1
  has_8 <- dfr$hs8
  dfr$d[has_1][get_d1[has_1]] <- TRUE
  dfr$d[has_8][get_d8[has_8]] <- TRUE
  dfr$hs1[has_1][get_d1[has_1]] <- FALSE
  dfr$hs8[has_8][get_d8[has_8]] <- FALSE
```

```
  ## individuals may recover/exit from health states 1, 2 & 7 only
  has_1 <- dfr$hs1
  has_2 <- dfr$hs2
  exit_2 <- dfr$exit_2
  has_7 <- dfr$hs7
  dfr$rhap[has_1][recover_hs1[has_1]] <- TRUE
  dfr$rhap[has_2][recover_hs2[has_2]] <- TRUE
  dfr$rcc[has_7][recover_hs7[has_7]] <- TRUE
  dfr$hs1[has_1][recover_hs1[has_1]] <- FALSE
  dfr$exit_2 <- FALSE                              # reset indicator for exiting Sepsis to FALSE every
time-step
  dfr$exit_2[has_2][recover_hs2[has_2]] <- TRUE   # set indicator to TRUE only in time-step when exiting
state 2
  dfr$hs2[has_2][recover_hs2[has_2]] <- FALSE
  dfr$hs7[has_7][recover_hs7[has_7]] <- FALSE

  ## individuals in state 7 may acquire HAP infection
  has_7 <- dfr$hs7
  dfr$hs1[has_7][get_hs1[has_7]] <- TRUE

  ## individuals may develop health state 2 if in origin state (state 1), and may progress
  ## to states 3, 4, 5, or 6 only in time-step following exiting state 2
  has_1 <- dfr$hs1
  has_2 <- dfr$hs2
  dfr$hs2[has_1][get_hs2[has_1]] <- TRUE
  dfr$hs3[exit_2][get_hs3[exit_2]] <- TRUE       # possibly move to state 3 only those who have just
exited state 2
  dfr$hs4[exit_2][get_hs4[exit_2]] <- TRUE       # same, for state 4
  dfr$hs5[exit_2][get_hs5[exit_2]] <- TRUE       # ... 5
  dfr$hs6[exit_2][get_hs6[exit_2]] <- TRUE       # ... 6
  dfr$hs8[has_7][get_hs8[has_7]] <- TRUE
  dfr$hs1[has_1][get_hs2[has_1]] <- FALSE        # exit SI if develop Sepsis
  dfr$hs7[has_7][get_hs8[has_7]] <- FALSE        # exit Diag/therapy if progress to Preterm/term

  ## Calculate combined DW  NB. 2:9 refers to column indices of 'has health-state-n' indicators
  dfr_hs_dw <- t(t(dfr[, 2:9]) * dw_all)
  dfr$dw <- 1 - rowProds(1 - dfr_hs_dw)
  dfr$dw.unadj <- rowSums(dfr_hs_dw)

  ## calculate total YLD (in days) over time, also stratified by disease and by state, using decomposition
method
  yld[i] <- get_yld(dfr)
   denom <- (dw1*dfr$hs1 + dw2*dfr$hs2 + dw3*dfr$hs3 + dw4*dfr$hs4 + dw5*dfr$hs5 + dw6*dfr$hs6 +
dw7*dfr$hs7 + dw8*dfr$hs8)
  yld_cc[i] <- sum(dfr$dw[dfr$hs7]*(dw7/denom[dfr$hs7])) + sum(dfr$dw[dfr$hs8]*(dw8/denom[dfr$hs8]))
  yld_hap[i] <- sum(dfr$dw[dfr$hs1]*(dw1/denom[dfr$hs1])) + sum(dfr$dw[dfr$hs2]*(dw2/denom[dfr$hs2])) +
                sum(dfr$dw[dfr$hs3]*(dw3/denom[dfr$hs3])) + sum(dfr$dw[dfr$hs4]*(dw4/denom[dfr$hs4])) +
                sum(dfr$dw[dfr$hs5]*(dw5/denom[dfr$hs5])) + sum(dfr$dw[dfr$hs6]*(dw6/denom[dfr$hs6]))
  yld_cc_state[1,i] <- sum(dfr$dw[dfr$hs7]*(dw7/denom[dfr$hs7]))
  yld_cc_state[2,i] <- sum(dfr$dw[dfr$hs8]*(dw8/denom[dfr$hs8]))
  yld_hap_state[1,i] <- sum(dfr$dw[dfr$hs1]*(dw1/denom[dfr$hs1]))
  yld_hap_state[2,i] <- sum(dfr$dw[dfr$hs2]*(dw2/denom[dfr$hs2]))
  yld_hap_state[3,i] <- sum(dfr$dw[dfr$hs3]*(dw3/denom[dfr$hs3]))
  yld_hap_state[4,i] <- sum(dfr$dw[dfr$hs4]*(dw4/denom[dfr$hs4]))
  yld_hap_state[5,i] <- sum(dfr$dw[dfr$hs5]*(dw5/denom[dfr$hs5]))
  yld_hap_state[6,i] <- sum(dfr$dw[dfr$hs6]*(dw6/denom[dfr$hs6]))

  yld_unadj[i] <- get_yld_unadj(dfr)
   denom <- (dw1*dfr$hs1 + dw2*dfr$hs2 + dw3*dfr$hs3 + dw4*dfr$hs4 + dw5*dfr$hs5 + dw6*dfr$hs6 +
dw7*dfr$hs7 + dw8*dfr$hs8)
  yld_cc_unadj[i] <- sum(dfr$dw.unadj[dfr$hs7]*(dw7/denom[dfr$hs7])) +
sum(dfr$dw.unadj[dfr$hs8]*(dw8/denom[dfr$hs8]))
  yld_hap_unadj[i] <- sum(dfr$dw.unadj[dfr$hs1]*(dw1/denom[dfr$hs1])) +
sum(dfr$dw.unadj[dfr$hs2]*(dw2/denom[dfr$hs2])) +
                sum(dfr$dw.unadj[dfr$hs3]*(dw3/denom[dfr$hs3])) +
sum(dfr$dw.unadj[dfr$hs4]*(dw4/denom[dfr$hs4])) +
                sum(dfr$dw.unadj[dfr$hs5]*(dw5/denom[dfr$hs5])) +
sum(dfr$dw.unadj[dfr$hs6]*(dw6/denom[dfr$hs6]))
  yld_cc_state_unadj[1,i] <- sum(dfr$dw.unadj[dfr$hs7]*(dw7/denom[dfr$hs7]))
  yld_cc_state_unadj[2,i] <- sum(dfr$dw.unadj[dfr$hs8]*(dw8/denom[dfr$hs8]))
  yld_hap_state_unadj[1,i] <- sum(dfr$dw.unadj[dfr$hs1]*(dw1/denom[dfr$hs1]))
  yld_hap_state_unadj[2,i] <- sum(dfr$dw.unadj[dfr$hs2]*(dw2/denom[dfr$hs2]))
  yld_hap_state_unadj[3,i] <- sum(dfr$dw.unadj[dfr$hs3]*(dw3/denom[dfr$hs3]))
  yld_hap_state_unadj[4,i] <- sum(dfr$dw.unadj[dfr$hs4]*(dw4/denom[dfr$hs4]))
  yld_hap_state_unadj[5,i] <- sum(dfr$dw.unadj[dfr$hs5]*(dw5/denom[dfr$hs5]))
  yld_hap_state_unadj[6,i] <- sum(dfr$dw.unadj[dfr$hs6]*(dw6/denom[dfr$hs6]))

  yld_cc_unadj[i] <- sum(dfr$hs7*dw7) + sum(dfr$hs8*dw8)
  yld_hap_unadj[i] <- sum(dfr$hs1*dw1) + sum(dfr$hs2*dw2) + sum(dfr$hs3*dw3) + sum(dfr$hs4*dw4) +
                      sum(dfr$hs5*dw5) + sum(dfr$hs6*dw6)
```

```
  yld_cc_state_unadj[1,i] <- sum(dfr$hs7*dw7)
  yld_cc_state_unadj[2,i] <- sum(dfr$hs8*dw8)
  yld_hap_state_unadj[1,i] <- sum(dfr$hs1*dw1)
  yld_hap_state_unadj[2,i] <- sum(dfr$hs2*dw2)
  yld_hap_state_unadj[3,i] <- sum(dfr$hs3*dw3)
  yld_hap_state_unadj[4,i] <- sum(dfr$hs4*dw4)
  yld_hap_state_unadj[5,i] <- sum(dfr$hs5*dw5)
  yld_hap_state_unadj[6,i] <- sum(dfr$hs6*dw6)

  ## record state-occupation over time, to calc prevalence[=no. individuals] per state over time (for
plotting)
  prev[i, ] <- colSums(dfr[, c(paste0("hs", 1:8), "d")])
 }
 return(list(prev,yld,yld_unadj,yld_cc,yld_cc_unadj,yld_hap,yld_hap_unadj,
             yld_cc_state,yld_cc_state_unadj,yld_hap_state,yld_hap_state_unadj))  # return also state-
specific yld
}


### Simulate
yld_total_m <- matrix(0,nrow=nsim,ncol=s)        # create matrix of (nsim x total time-steps)
yld_total_unadj_m <- matrix(0,nrow=nsim,ncol=s)  # create matrix of (nsim x total time-steps)
yld_cc_m <- matrix(0,nrow=nsim,ncol=s)          # create matrix of (nsim x total time-steps)
yld_hap_m <- matrix(0,nrow=nsim,ncol=s)
yld_cc_unadj_m <- matrix(0,nrow=nsim,ncol=s)
yld_hap_unadj_m <- matrix(0,nrow=nsim,ncol=s)

yld_cc_state_m <- array(0,dim=c(nsim,2,s))       # 2 states
yld_cc_state_unadj_m <- array(0,dim=c(nsim,2,s))
yld_hap_state_m <- array(0,dim=c(nsim,6,s))      # 6 states
yld_hap_state_unadj_m <- array(0,dim=c(nsim,6,s))


# For totals over entire simulation period:
yld_total_median_95pc <- matrix(0,nrow=1,ncol=3)
yld_total_unadjusted_median_95pc <- matrix(0,nrow=1,ncol=3)
yld_cc_median_95pc <- matrix(0,nrow=1,ncol=3)
yld_cc_unadj_median_95pc <- matrix(0,nrow=1,ncol=3)
yld_hap_median_95pc <- matrix(0,nrow=1,ncol=3)
yld_hap_unadj_median_95pc <- matrix(0,nrow=1,ncol=3)

yld_cc_state_median_95pc <- array(0,dim=c(2,3))
yld_cc_state_unadj_median_95pc <- array(0,dim=c(2,3))
yld_hap_state_median_95pc <- array(0,dim=c(2,3))
yld_hap_state_unadj_median_95pc <- array(0,dim=c(2,3))


dd <- 365    # units days to years
returnList0 <- rlply(nsim, sim(), .progress = "text")

for(m in 1:nsim) {
 returnList <- returnList0[[m]]

 prev <- returnList[[1]]               # prevalence is from the last run only
 yld_total_m[m,] <- returnList[[2]]/dd
 yld_total_unadj_m[m,] <- returnList[[3]]/dd
 yld_cc_m[m,] <- returnList[[4]]/dd
 yld_cc_unadj_m[m,] <- returnList[[5]]/dd
 yld_hap_m[m,] <- returnList[[6]]/dd
 yld_hap_unadj_m[m,] <- returnList[[7]]/dd
 yld_cc_state_m[m,,] <- returnList[[8]]/dd
 yld_cc_state_unadj_m[m,,] <- returnList[[9]]/dd
 yld_hap_state_m[m,,] <- returnList[[10]]/dd
 yld_hap_state_unadj_m[m,,] <- returnList[[11]]/dd
}

# Calculate median and 95% UI from nsim iterations:
yld_total_median_95pc <- quantile(apply(yld_total_m,1,sum),probs=c(0.025,0.5,0.975))
yld_total_unadj_median_95pc <- quantile(apply(yld_total_unadj_m,1,sum),probs=c(0.025,0.5,0.975))
yld_cc_median_95pc <- quantile(apply(yld_cc_m,1,sum),probs=c(0.025,0.5,0.975))
yld_cc_unadj_median_95pc <- quantile(apply(yld_cc_unadj_m,1,sum),probs=c(0.025,0.5,0.975))
yld_hap_median_95pc <- quantile(apply(yld_hap_m,1,sum),probs=c(0.025,0.5,0.975))
yld_hap_unadj_median_95pc <- quantile(apply(yld_hap_unadj_m,1,sum),probs=c(0.025,0.5,0.975))
yld_cc_state_median_95pc <- t(apply(apply(yld_cc_state_m,c(1,2),sum),2,quantile,probs=c(0.025,0.5,0.975)))
yld_cc_state_unadj_median_95pc <-
t(apply(apply(yld_cc_state_unadj_m,c(1,2),sum),2,quantile,probs=c(0.025,0.5,0.975)))
yld_hap_state_median_95pc <-
t(apply(apply(yld_hap_state_m,c(1,2),sum),2,quantile,probs=c(0.025,0.5,0.975)))
yld_hap_state_unadj_median_95pc <-
t(apply(apply(yld_hap_state_unadj_m,c(1,2),sum),2,quantile,probs=c(0.025,0.5,0.975)))
```
